# One-Step Synthesis of Pt/Graphene Composites from Pt Acid Dissolved Ethanol via Microwave Plasma Spray Pyrolysis

**DOI:** 10.1038/srep33236

**Published:** 2016-09-13

**Authors:** Eun Hee Jo, Hankwon Chang, Sun Kyung Kim, Ji-Hyuk Choi, Su-Ryeon Park, Chong Min Lee, Hee Dong Jang

**Affiliations:** 1 Rare Metals Research Division, Korea Institute of Geoscience & Mineral Resources, Deajeon, 34132, Korea; 2 Department of Nanomaterials Science and Engineering, University of Science & Technology, Deajeon, 34113, Korea

## Abstract

Pt nanoparticles-laden graphene (Pt/GR) composites were synthesized in the gas phase from a mixture of ethanol and Pt precursor by microwave plasma spray pyrolysis. The morphology of Pt/GR composites has the shape of wrinkled sheets of paper, while Pt nanoparticles (Pt NPs) that are less than 2.6 nm in the mean diameter are uniformly well deposited on the surface of GR sheets stacked in only three layers. The Pt/GR composite prepared with 20 wt% of Pt had the highest specific surface area and electrochemical surface area of up to 402 m^2^ g^−1^ and 77 m^2^ g^−1^ (Pt), respectively. In addition, the composite showed superior electrocatalytic activity compared with commercial Pt-carbon black. The excellent electrocatalytic activity was attributed to the high specific surface area and electrochemical surface area of the Pt/GR composite directly produced by microwave plasma spray pyrolysis. Thus, it is clearly expected that the Pt/GR composite is a promising material for DMFC catalysts.

Graphene (GR), a single atomic layer of graphite, has drawn much attention due to its superior physical and chemical properties, large surface area, and excellent electronic conductivity, which makes it promising for potential applications in various fields, such as nanocomposites^1^,^2^, solar cells^3^,^4^, biosensors^5^,^6^, supercapacitors^7^, and fuel cells^8^. Recently, graphene supporting metal nanocomposites with enhanced electrical conductivity have often been used as promising catalyst carriers for energy conversion devices, e.g. direct methanol fuel cells (DMFCs)^8^,^9^,^10^. In particular, graphene supporting Pt nanoparticles (Pt/GR) have been investigated as good catalysts for DMFCs.

Many studies have been reported on the preparation of Pt/GR composites^11^,^12^,^13^,^14^,^15^,^16^,^17^. A few research groups have synthesized the Pt nanoparticles (Pt NPs) onto GR sheets by reducing Pt ions in a liquid-phase system. The GR decorated with Pt NPs was mainly reduced by a chemical reductant (e.g. hydrazine, H_2_O_2_) or thermal treatment in an Ar/H_2_ atmosphere. However, all the methods to make the Pt/GR composites are time consuming due to many kinds of chemical reaction steps and long reaction time. Also, this graphene oxide (GO) reduction process can introduce many structural defects from the oxidation process for GR synthesis. Moreover, the residual oxygen groups in the reduced GO can cause poor electrical conductivity and limit its further applications. In our previous study, we synthesized Pt NPs-laden graphene crumples from a colloidal mixture of GO and platinic acid via a one-step aerosol process^18^. This method was a facile and continuous process. There was also a very short reaction time of several seconds and no post-heat treatment was needed. However, the usage of the GO colloidal solution prepared by Hummer’s method from graphite powder led to the same structural defects when synthesizing Pt/GR composites. In addition, it is not easy to synthesize Pt/GR with single and bilayer graphene^19^, which can result in a reduced specific surface area and electrochemical surface area.

Very recently, Dato *et al*. reported an approach to synthesize high quality graphene from ethanol in the gas phase by using a substrate-free, atmospheric microwave plasma reactor^20^. Also, Tatarova *et al*. synthesized graphene sheets that have a different morphology by adjusting the outlet gas temperature of microwave plasma^21^. According to previous studies, the microwave plasma method is a fast and continuous process with no contamination of the resultant products. In addition, graphene sheets were synthesized directly from carbon based organic compounds in the gas phase while the entire production process took place in fractions of a second in an atmospheric-pressure environment. Thus, it is also expected that metal nanoparticle decorated GR can be synthesized from a mixture of ethanol and Pt precursor via the microwave plasma spray pyrolysis.

In this study, we present for the first time a one-step synthesis of Pt nanoparticle laden GR in the gas phase as the electrochemical catalyst for DMFCs by the microwave plasma spray pyrolysis. The precursor droplets of ethanol and chloroplatinic acid (H_2_PtCl_6_) pass through the microwave argon plasma, where the decomposition of ethanol and Pt precursor takes place and then the Pt/GR chemistry reacts. The effect of Pt content in GR sheets on the morphology, crystal structure, specific surface area and electrochemical surface area of Pt/GR was investigated in order to characterize the material properties of composites. We also compared methanol electrochemical oxidation at the as-prepared Pt/GR composites and commercial Pt/C, and confirmed that the former showed better electrocatalytic properties.

## Results

**Figure 1:**
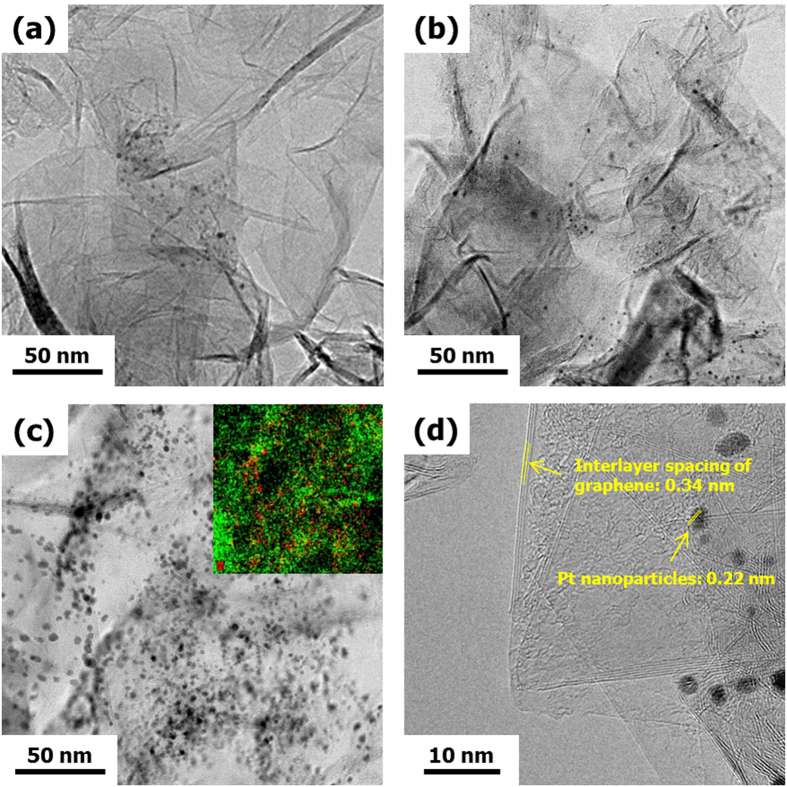
TEM images of Pt/GR composites prepared at different Pt concentration of (**a**) 5 wt%, (**b**) 10 wt%, and (**c**) 20 wt%. (Inset of (**c**) is the TEM-EDS mapping image). (**d**) HRTEM image of Pt/GR composite.

In order to determine the content of Pt in the Pt/GR composites, the thermos gravimetric analysis (TGA) was conducted in a temperature range from 25 to 1000 °C. The TGA result could confirm that the mass fraction of Pt NPs in the composites was the same as the pre-determined fraction in the precursor solution (Figure S1). Figure 1 shows the transmission electron microscope (TEM) images of the Pt/GR composite prepared with various Pt contents of (a) 5, (b) 10, and (c) 20 wt% via microwave plasma spray pyrolysis. The morphology of the Pt/GR composite was the shape of a wrinkled sheet of paper. The TEM images indicate that Pt NPs are uniformly supported on the surface of GR sheets. Higher numbers of Pt NPs are observed on the GR at higher Pt contents. The average particle size of Pt NPs increased from approximately 1.22 to 2.60 nm in diameter when the Pt concentration increased (Figure S3). The inset of Fig. 1(c) is a TEM-energy dispersive X-ray spectroscopy (EDS) mapping image of the Pt/GR composite prepared with 20 wt% of Pt content. The red color in the Figure indicates the Pt NPs. It is also observed that the Pt NPs were uniformly deposited on the GR sheets. Moreover, the high-resolution TEM (HRTEM, Fig. 1(d)) image clearly shows the layered crystal structure of the GR sheets, with an interlayer spacing of 0.34 nm, which is larger than that of the (002) plane of graphite (0.33 nm). The lattice fringe of the nanoparticle has an interlayer spacing of 0.22 nm and is ascribed to the (111) plane of Pt. These crystal structures of Pt and GR sheets were further confirmed by X-ray diffractometry (XRD).

**Figure 2:**
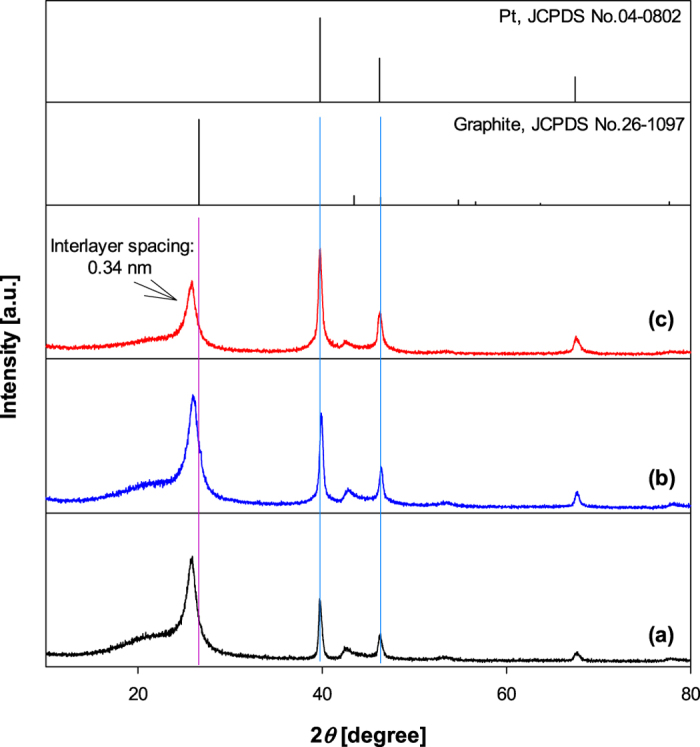
XRD patterns of Pt/GR composite prepared at different Pt concentrations of (**a**) 5 wt%, (**b**) 10 wt% and (**c**) 20 wt%.

Figure 2 shows the XRD patterns of the as-prepared Pt/GR composites. The standard card of graphite (JCPDS No. 26-1097) showed the most intense (002) peak at 26.5^o^, while the diffraction peaks of the C (002) plane can be clearly observed at 2*θ* around 25.5^o^ for the Pt/GR composites. The interlayer spacing (d_002_) was calculated using Bragg’s law^22^. The (002) peaks appearing at 25.5^o^ for Pt/GR composites corresponds to an interlayer spacing (d_002_) of 0.34 nm. This interlayer spacing of GR sheets is in good agreement with the HRTEM results above. Also, the diffraction peaks of all samples located at 39.81^o^, 46.09^o^, and 67.79^o^ could be ascribed to (111), (200), and (220) crystalline planes of Pt^12^, which corresponded well to JCPDS No. 04-0802. Therefore, these results show that the Pt/GR composites are successfully synthesized by microwave plasma spray pyrolysis. Meanwhile, when the Pt concentration increased, the intensity of the Pt peak also increased due to a high Pt content in the composites. This is also in accordance with the TEM result.

**Figure 3:**
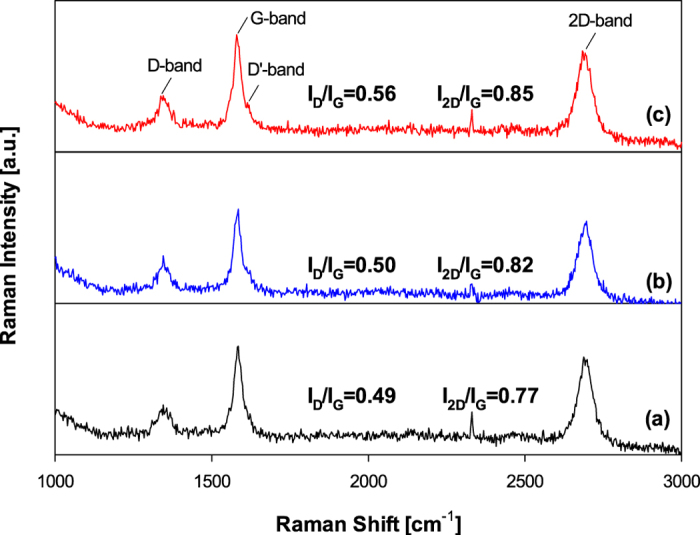
Raman spectra of Pt/GR composite prepared at different Pt contents of (**a**) 5 wt%, (**b**) 10 wt%, and (**c**) 20 wt%.

Figure 3 shows the Raman spectra of Pt/GR composites measured at an excitation wave of 532 nm under ambient conditions. Figure 3(a–c) presents two characteristic G- and 2D-bands of GR at 1580 and 2680 cm^−1^. The band at 1350 cm^−1^ is assigned to the D-band, which is due to the A_1g_ symmetry in a structural defect or partially disordered structures of the sp^2^ domains in GR sheets^23^. The peak at 1610 cm^−1^ is called the D’-band, which occurs in the presence of defects^24^. Furthermore, the relative intensity of D- and G-bands is a convenient way to evaluate the extent of defects and the size of the in-plane sp^2^ domain in the GR sheets^25^. The intensity ratio of the I_D_/I_G_ in the GR sheets increases from 0.49 to 0.56 as the Pt content increases from 5 wt% to 20 wt%. The increased I_D_/I_G_ ratio of GR sheets may be due to the high density of the defects on the structure of the GR sheets. Also, the intensity ratios are much lower than those obtained from chemically reduced graphite oxide in a liquid-phase system^12^,^18^,^19^. Meanwhile, the integrated intensity ratio of 2D to the G band (I_2D_/I_G_) can be used to determine the number of layers of GR sheets^26^. As shown in Fig. 3, the intensity ratios of the composites are about ~0.8. These results show that only three layer GR sheets exist in all the as-prepared Pt/GR composites. In the case of the GR sheets synthesized by chemical reduction in a liquid phase, the sheets tended to easily restack and aggregate due to a strong van-der Waals attraction. According to the Raman and XRD results, it is noted that the structural defect of the as-prepared GR was less than the graphene obtained by other methods.

**Figure 4:**
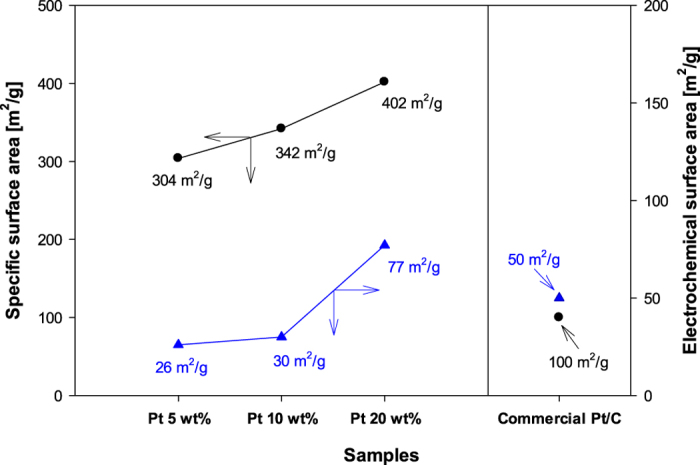
Specific surface area and electrochemical surface area of commercial Pt/C and Pt/GR composite prepared with various Pt contents of 5 wt%, 10 wt%, and 20 wt%.

The specific surface area (SSA) and electrochemical surface area (ECSA) of the as-prepared composites are shown in Fig. 4. The SSA for the Pt/GR composites are 304, 342, and 402 m^2^ g^−1^, corresponding to Pt loading of 5 wt%, 10 wt% and 20 wt%, respectively. In case of the GR sheets without Pt nanoparticles, the SSA exhibits 1318 m^2^ g^−1^ (Figure S2, Table S1). Although these values are lower than the theoretical SSA of 2630 m^2^ g^−1^ for individual isolated graphene sheets^27^, the SSAs of microwave produced Pt/GR composites are much higher than those of previously reported Pt/GR composites (146 m^2^ g^−1^) synthesized by a one-step aerosol process coupled with Hummer’s method^28^. The greatly increased SSAs are due to their thin-layered structure, as shown in Raman analysis. In addition, the SSAs of the composites increase dramatically with increasing Pt contents. The increase of the SSA is attributed to the increase in the number of Pt NPs on the surface of GR sheets. The high SSAs of Pt/GR composites are beneficial to understand the mass activity of Pt based catalysts and mass transport of electrolytes and methanol in the electric catalytic process^29^. Furthermore, the ECSA of the Pt/GR composites is important to measure the catalytic performance of Pt catalysts. Therefore, we calculated the ECSA from the cyclic voltammogram measurements (Figure S4) by using the following equation^12^:


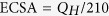


where Q_H_ represents the charge of the hydrogen underpotential desorption (μC), and 210 represents the charge density of H monolayer on a bright Pt (μC cm^−2^). The ECSAs of the as-prepared Pt/GR composites are 25, 30, and 77 m^2^ g^−1^ (Pt), respectively. These values have a similar trend to those of the SSA of the Pt/GR composites. Moreover, the higher ECSA values are obtained at a higher content of Pt. This is due to the deposition of the higher loading of Pt on the electrode. In particular, the SSA and ECSA of the Pt/GR composite prepared with 20 wt% of Pt are higher than those of the other Pt/GR composites. These results indicate that the Pt/GR composite with a higher loading of Pt provides more electrocatalytic active sites; thus, we believe that the composite will have superior ability in regards to the electrochemical methanol reactions. A commercial Pt/carbon black catalyst (Pt 20 wt%, Alfa Aesar, USA) was used as a reference for comparison. The SSA and ECSA of the as-prepared Pt/GR composite with 20 wt% of Pt are also higher than those of the commercial Pt/C.

**Figure 5:**
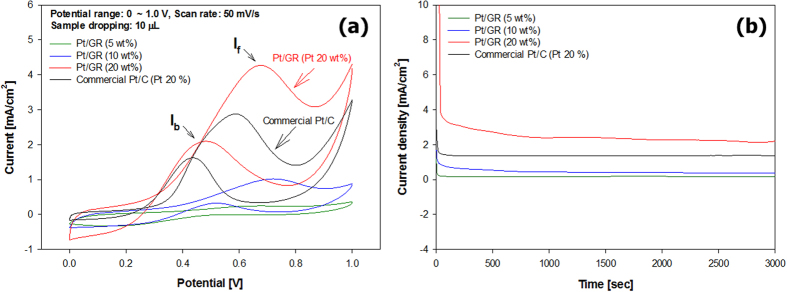
(**a**) CVs for methanol oxidation reaction catalyzed by commercial Pt/Carbon black and Pt/GR composite in the mixture solution of 0.05 M H_2_SO_4_ + 1 M CH_3_OH within the potential range of 0–1.0 V (**b**) Chronoamperometry employed by the Pt/Carbon black and Pt/GR composite with different Pt contents of 5, 10, and 20 wt% at 0.4 V in a 0.5 M H_2_SO_4_ and 1 M CH_3_OH.

The as-prepared Pt/GR composites were tested for the oxidation of methanol as a DMFC electrocatalyst. The cyclic voltammetry (CV) measurement of the methanol oxidation reaction was examined with Pt/GR composites with different Pt loadings. As shown in Fig. 5a, the intensities of the electrocatalytic current increase with an increasing Pt content in the Pt/GR, indicating an enhanced methanol oxidation at increased active surface area. Compared with the Pt/GR composites prepared with 5 and 10 wt% of Pt and the commercial Pt/C, the Pt/GR prepared with 20 wt% of Pt exhibits highest electrocatalytic current. The ratio of the forward peak current density (I_f_) to the reverse anodic (backward) peak current density (I_b_), i.e., I_f_/I_b_, is obtained from the CVs of the methanol oxidation reaction. The reverse anodic peak for the methanol oxidation reaction is attributed to the removal of the incompletely oxidized carbonaceous species formed in the forward scan. Hence, the ratio (I_f_/I_b_) can indicate the efficiency of the oxidation of methanol in the forward potential scan. The higher I_f_/I_b_ ratio indicates less poisoning species forming and therefore methanol can be oxidized to carbon dioxide more efficiently^14^. It is clearly shown in Fig. 5a that the Pt/GR prepared at 20 wt% Pt content exhibits superior electrocatalytic activity towards the oxidation of methanol than commercial Pt/C. The higher activity of the Pt/GR composite was resulted from the catalyst having a high SSA and ECSA of the catalysts, which originated from a combination of densely distributed small Pt nanoparticles and high conductivity of wrinkled GR sheets. In addition, a much higher I_f_/I_b_ value (2.2) from the Pt/GR composite demonstrates that more effective oxidation of methanol occurs on the Pt/GR composite in the forward peak current density, forming a less poisoning species compared with commercial Pt/C (1.7). Chronoamperometry was measured at a fixed potential of 0.4 V for 50 min to compare the long-term performance of the catalysts in the methanol oxidation reaction (Fig. 5b). It was measured that the current density and stability of methanol oxidation reaction with the Pt/GR prepared at 20 wt% Pt were higher than those with the commercial Pt/C.

**Figure 6:**
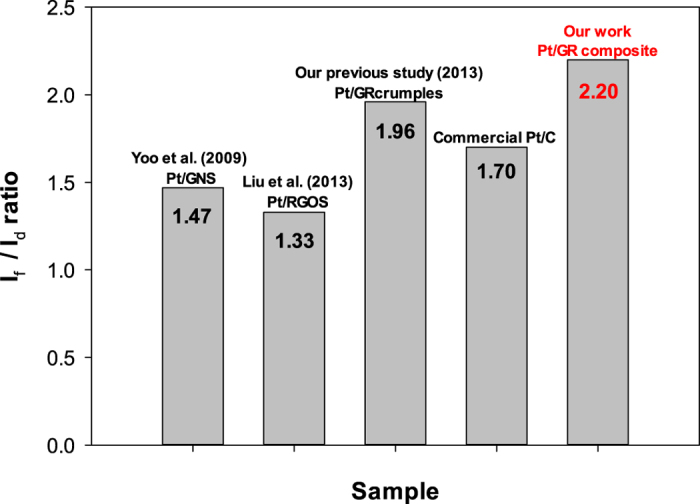
Comparison of catalysts activity of commercial Pt/C and Pt/GR composites with those reported in previous studies.

Figure 6 shows a comparison of the Pt/GR catalytic activity normalized by Pt loading reported in previous studies. Yoo *et al*. prepared Pt/graphene nanosheets (Pt/GNS) catalysts by thermal treatment, of which the I_f_/I_b_ ratio was 1.47 for the methanol oxidation reaction^14^. Liu *et al*. found that the I_f_/I_b_ ratio of their Pt/reduced graphene oxide nanoscroll (Pt/RGOS) prepared by ultrasonication using H_2_O_2_ was 1.33 for the methanol oxidation reaction^12^. In our previous study, Pt/GR crumples synthesized by a one-step aerosol process had an I_f_/I_b_ ratio of 1.96^18^. Compared with the previous results of Pt/GR, the microwave produced Pt/GR catalysts in this study had the highest I_f_/I_b_ ratio. Meanwhile, all of the Pt/GR catalysts in previous studies were synthesized from graphene oxide (GO) sheets prepared by chemical oxidation process. The sizes of Pt/GR catalysts are more than 1 μm as shown in their SEM images. In this work, the size of as-prepared Pt/GR composites having wrinkled morphology is about ~200 nm. The smaller size of Pt/GR composites leads to more edges with active sites, which also enhance the catalytic activity.

## Discussion

Pt nanoparticles laden GR sheets were successfully fabricated from a mixture solution of ethanol and Pt precursor via a continuous microwave plasma spray pyrolysis. As the Pt contents in the Pt/GR composites increased, the SSAs and ECSAs also increased. In particular, the Pt/GR composite prepared with 20 wt% of Pt had the highest SSA and ECSA, which was due to the very thin GR sheets of three layers and uniform deposition of Pt NPs on the GR sheets. The Pt/GR composite also showed a superior catalytic performance for methanol oxidation than that of the commercial Pt/carbon black. The excellent performance was attributed to the large SSA and ECSA of Pt/GR composites, small nanoparticle size and their uniform distributions. It is confirmed that the microwave plasma spray pyrolysis is a promising process to synthesize Pt/GR composites for the enhanced DMFC catalysts.

## Methods

### Synthesis of Pt/GR composite

**Figure 7:**
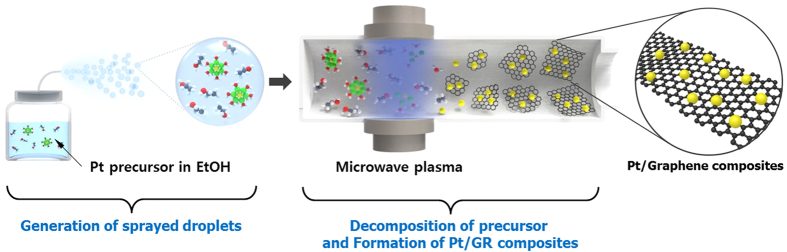
Schematic diagram of a microwave plasma spray pyrolysis.

Pt/Graphene (Pt/GR) composites were synthesized via microwave plasma spray pyrolysis^20^. Chloroplatinic acid hexahydrate (H_2_Cl_6_Pt∙6H_2_O, Sigma Aldrich, ≥ 37.5% Pt basis), and ethanol (C_2_H_5_OH, Samchun, 99.9%) were used as the precursor and organic solvent of Pt/GR composites. The concentration of Pt was 0.1, 0.2, and 0.4 wt%, respectively, in a colloidal mixture. Figure 7 shows the schematic diagram of a microwave plasma spray pyrolysis. The system consists of an ultrasonic nebulizer for the feeding of precursor droplets, a plasma reactor for the synthesis of Pt/GR composites, and a filter for the particles sampling. The experiments were carried out in an atmospheric pressure reactor. The argon gas (2 L min^−1^) as working and swirl gas was used to generate argon plasma. The microwave was provided by a 2.45 GHz generator, whose output power was 1.4 kW. In the microwave plasma aerosol reactor, micron-sized droplets were generated by an ultrasonic nebulizer from the mixture solution of Pt precursor and ethanol. Then, the droplets were carried into the plasma reactor by Ar gas at 1 L min^−1^. The droplets had a residence time on the order of 0.74 s inside the plasma. During this brief time, the mixture solution of the Pt precursor and ethanol rapidly evaporated and decomposed in the plasma, which formed a Pt/GR solid powder. The as-prepared products were collected on a filter sampler. For further enhancement of Pt crystallinity, the as-prepared Pt/GR composites were thermal-treated at 800 °C for 10 min under an Ar atmosphere.

### Analysis

The morphologies and elemental composition of the as-prepared Pt/GR composite were observed with a transmission electron microscope (TEM; JEM-ARM200F, JEOL, Japan) and an energy dispersive X-ray spectroscopy (EDS; Quantax 400, Bruker, UK). The crystallinity of the Pt/GR composite was analyzed with X-ray diffractometry (XRD; RTP 300 RC, Rigaku, Japan). Raman spectra were obtained using a Raman spectrometer (Dimension-P1, Lambda Solution, Inc., USA) with an argon-ion continuous-wave laser (532 nm) as the excitation source. The specific surface area (SSA) of the samples was measured using a nitrogen adsorption analyzer (BET; Tristar 3000, Micromeritics, USA). As-prepared samples were first degassed in the sample degas system (VacPrep 061, Micromeritics, USA) at 200 °C for 4 h prior to the analysis. The SSA was calculated using the multipoint BET method on six points of the adsorption isotherm in the relative pressure (P/P_0_) range from 0.05 to 0.3^30^. The thermos gravimetric analysis (TGA) was conducted to measure the Pt contents in the composites at a temperature range from 25 to 800 °C with a heating rate of 5 °C min^−1^ under air (DTG-60H, Shimadzu, Japan).

### Electrochemical measurements

Electrochemical measurements were conducted at room temperature with the as-prepared Pt/GR modified working electrodes with an electrochemical interface instrument (VSP, Bio-Logics, USA). The as-prepared Pt/GR (8 mg) composites were dispersed in a mixed solution containing deionized water (1.78 mL), ethanol (0.2 mL), and Nafion (Sigma Aldrich, 5%, 0.02 mL). Ten microliters of the Pt/GR colloid were dropped onto the surface of a glassy carbon electrode (GCE) and left to dry at room temperature. The cyclic voltammogram (CV) measurements of the methanol electro-oxidation were carried out in a mixed solution of 0.05 M H_2_SO_4_ + 1 M CH_3_OH within a potential range of 0–1.0 V (vs. SCE) with a scan rate of 50 mV s^−1^. A conventional three-electrode cell was used with a glassy carbon electrode (diameter: 3 mm) as the working electrode, an Ag/AgCl electrode as the reference electrode, and a platinum foil as the counter electrode.

## Additional Information

**How to cite this article**: Jo, E. H. *et al*. One-Step Synthesis of Pt/Graphene Composites from Pt Acid Dissolved Ethanol via Microwave Plasma Spray Pyrolysis. *Sci. Rep.* **6**, 33236; doi: 10.1038/srep33236 (2016).

## Supplementary Material

Supplementary Information
