# Supplementary material for: One-Step Synthesis of Pt/Graphene Composites from Pt Acid Dissolved Ethanol via Microwave Plasma Spray Pyrolysis
Source: Sci Rep. 2016 Sep 13;6:33236. doi: 10.1038/srep33236 (PMC5020682; doi:10.1038/srep33236)
Supplement: Supplementary Information [file srep33236-s1.pdf]

Supplementary Information

One-Step Synthesis of Pt/Graphene Composites from Pt Acid Dissolved Ethanol via Microwave Plasma Spray Pyrolysis

Eun Hee Jo[1,2,†], Hankwon Chang[1,2,†], Sun Kyung Kim[1], Ji-Hyuk Choi[1], Su-Ryeon Park[1,2], Chong Min Lee[1,2], Hee Dong Jang[1,2,*]

[1]Rare Metals Research Division, Korea Institute of Geoscience & Mineral Resources, Deajeon, 34132, Korea

[2]Department of Nanomaterials Science and Engineering, University of Science & Technology, Deajeon 34113, Korea

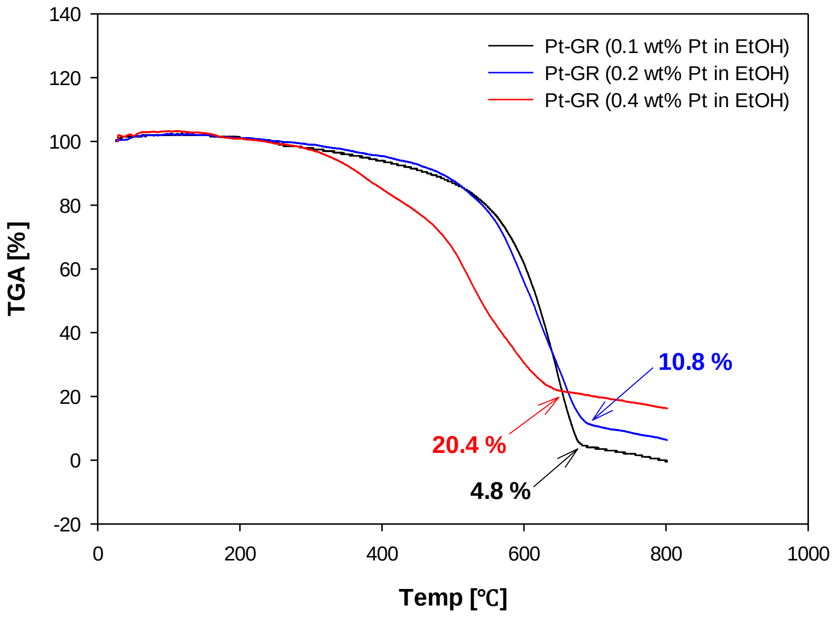

Figure S1 | TGA curves of Pt/GR composite prepared at different concentration of chloroplatinic acid hexahydrate in ethanol.

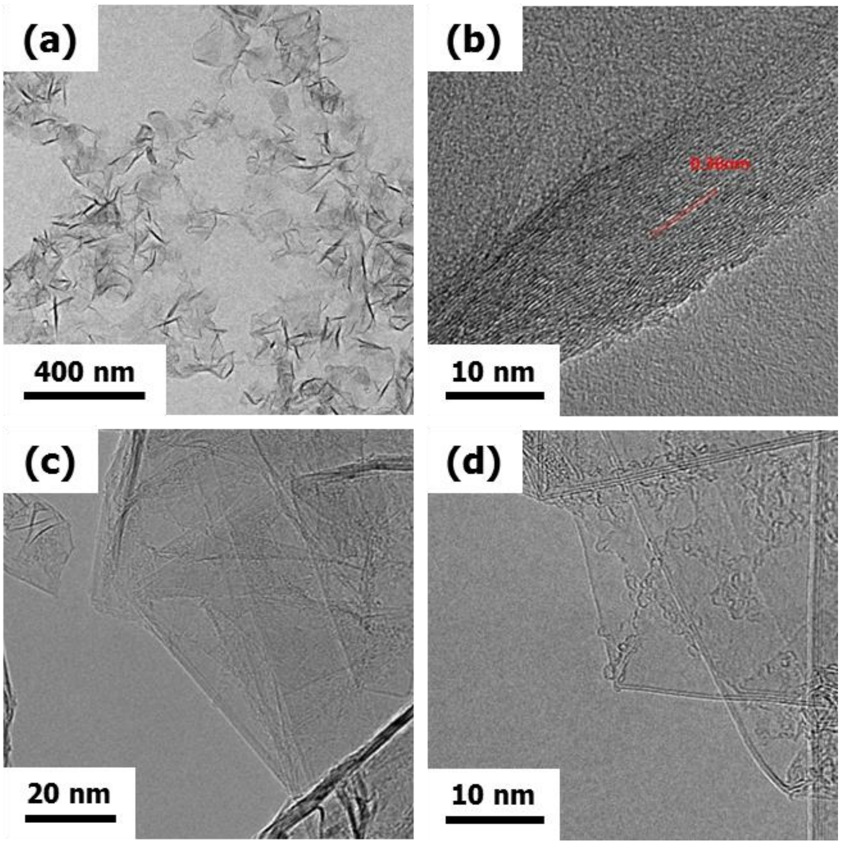

Figure S2 | TEM images of GR sheets prepared by ethanol via microwave plasma spray pyrolysis.

Table S1 | Specific surface area of GR sheets prepared by ethanol via microwave plasma spray pyrolysis.

Sample	Specific surface area (m^2/g)
GR sheets	1318

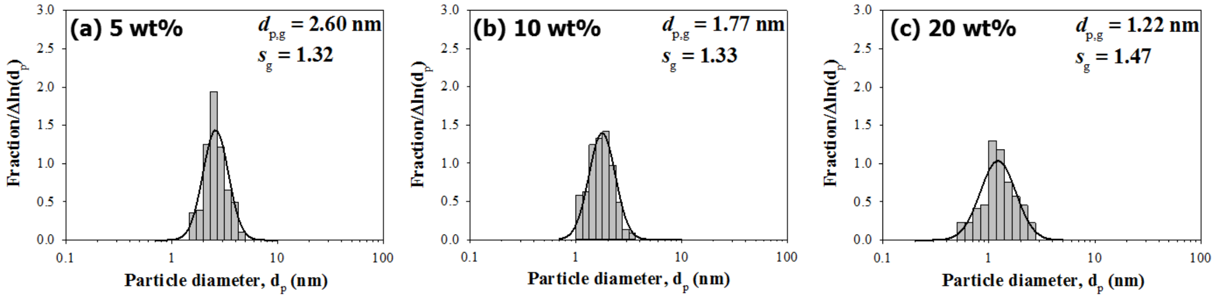

Figure S3 | Size distributions of Pt/GR composite prepared at different Pt contents of (a) 5 wt%, (b) 10 wt%, and (c) 20 wt%.

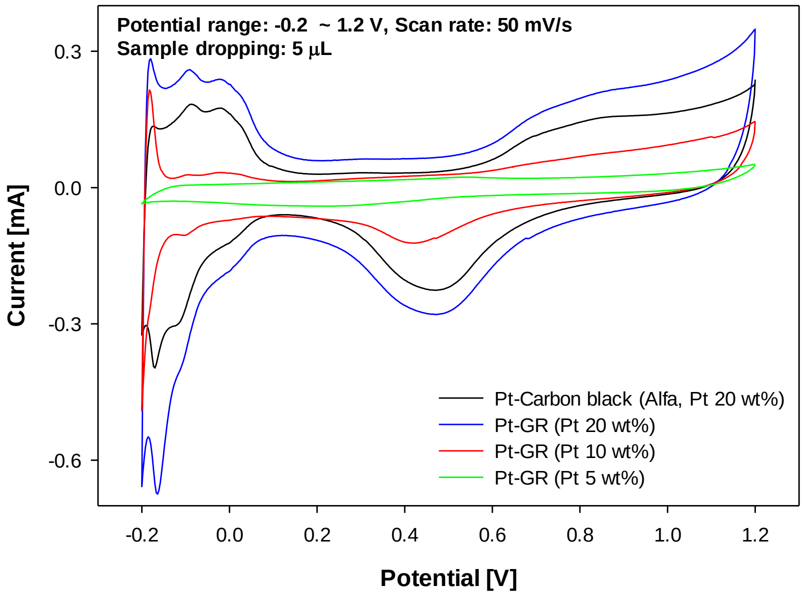

Figure S4 | Cyclic voltammograms of the Pt/Carbon black and Pt/GR composite in a 0.5 M H_2SO_4 solution.

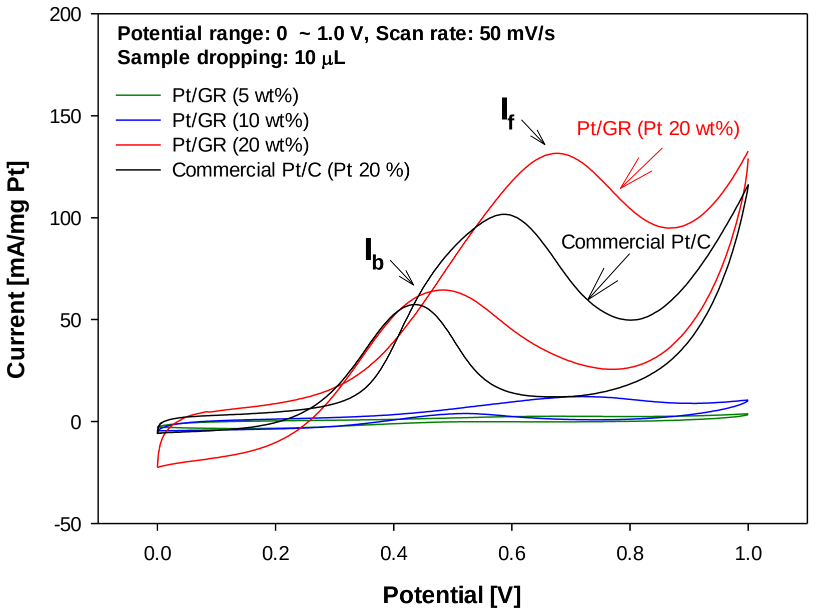

Figure S5 | CVs of Pt mass normalized methanol oxidation reaction on commercial Pt/Carbon black and Pt/GR composite measured in the mixture solution of 0.05 M H_2SO_4 + 1 M CH_3OH within the potential range of 0 - 1.0 V at a rate of 50 mV/s.

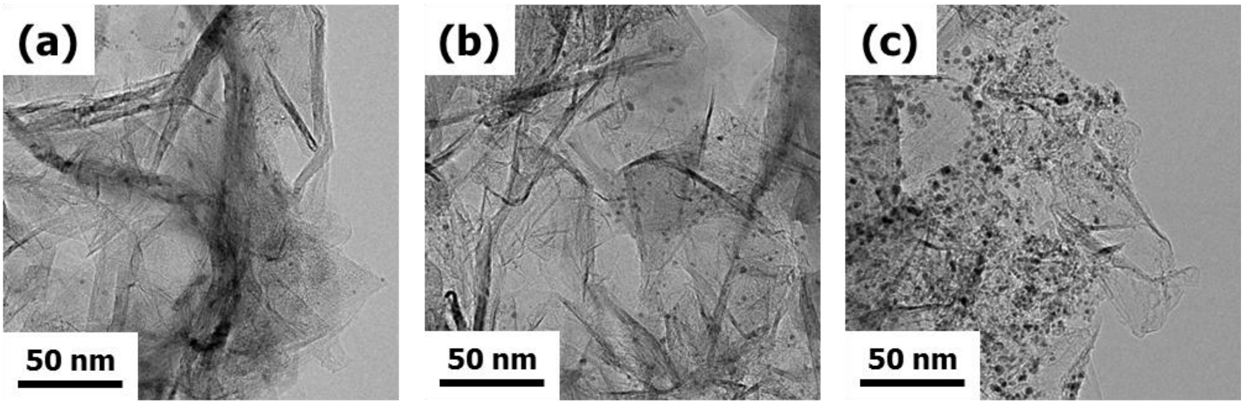

Figure S6 | TEM images of Pt/GR composites prepared at different Pt concentration of (a) 5 wt%, (b) 10 wt%, and (c) 20 wt% after long-term test.

Table S2 | Comparison of catalytic activity of current Pt/GR and other reported Pt based precious catalysts.

**Sample**	**I_f/I_b ratio**
Pt/C (Kim et al. 2015, Jang et al. 2013, our work)	1.02, 1.30, 1.70
Pt-Ag/C (Kim et al. 2015)	1.29
Pt-Ru/C (Wei et al. 2011)	2.10
Pt-Au (Dutta et al. 2015)	2.15
Pt-Pd (Dutta et al. 2015)	2.45
Pt/GR (our work)	2.20

References

1. Kim, S. M. et al. Characterizatio and electrocatalytic activity of Pt-M (M=Cu, Ag, and Pd) bimetallic nanoparticles synthesized by pulsed plasma discharge in water. *J. Nanopart. Res.***17**, 284 (2015).

2. Jang, H. D. et al. One-step synthesis of Pt-nanoparticles-laden graphene crumples by aerosol spray pyrolysis and evaluation of their electrocatalytic activity. *Aerosol Sci. Technol.* **47**, 93-98 (2013).

3. Wei, Y. C. et al. Promotion of Pt-Ru/C catalysts driven by heat treated induced surface segregation for methanol oxidation reaction. *Jouranl of Alloys and Compounds* **509**, 535-541 (2011).

4. Dutta, S. et al. Facile Synthesis of Bimetallic Au-Pt, Pd-Pt, and Au-Pd Nanostructures: Enhanced Catalytic Performance of Pd-Pt Analogue towards Fuel Cell Application and Electrochemical Sensing. *Electrochim. Acta* **180**, 1075-1084 (2015).
